# Auditory Sentence Processing in Bilinguals: The Role of Cognitive Control

**DOI:** 10.3389/fpsyg.2020.00898

**Published:** 2020-05-27

**Authors:** Niloofar Akhavan, Henrike K. Blumenfeld, Tracy Love

**Affiliations:** ^1^School of Speech, Language, and Hearing Sciences, San Diego State University, San Diego, CA, United States; ^2^Center for Research in Language, University of California, San Diego, San Diego, CA, United States; ^3^Joint Doctoral Program in Language and Communicative Disorders, San Diego State University/University of California, San Diego, San Diego, CA, United States

**Keywords:** online sentence processing, bilingualism, cognitive control, eye-tracking, similarity-based interference

## Abstract

A number of research studies have shown that the unique need in bilinguals to manage both of their languages positively impacts their cognitive control processes. Yet, due to a dearth of studies at the sentence level, it is still unclear if this benefit extends to sentence processing. In monolinguals and bilinguals, cognitive control helps in reinterpretation of garden path sentences but it is still unknown how it supports the real-time resolution of interference during parsing, such as the type of interference seen in the processing of object relative (OR) sentences. In this study, we compared monolinguals and bilinguals during online spoken OR sentence processing and examined if both groups used cognitive control to resolve interference. In this eye-tracking visual world (ETL-vw) study, OR sentences were aurally presented to 19 monolingual and 21 Spanish-English bilingual adults while gaze patterns were captured throughout the time course of the sentence. Of particular interest was the post-verb position, where the listener connects the verb to its direct object. In OR constructions (e.g., “The man that the boy pushes__ has a red shirt.”), the verb (‘pushes’) links to its syntactically licensed direct object (‘the man’) at verb offset. During syntactic linking, the parser crosses over an intervening noun phrase (NP, ‘the boy’) and the two NP activations create interference. The nature of this paradigm allows us to measure interference and its resolution between the intervening NP and the displaced object in real-time. By relating sentence processing patterns with cognitive control measures, high- and no- conflict N-Back tasks, we investigated group differences in the use of cognitive control during sentence processing. Overall, bilinguals showed less interference than monolinguals from the intervening NP during the real time processing of OR sentences. This interference effect and its resolution was significantly predicted by cognitive control skills for bilingual, but not monolingual listeners. This enhanced effect in bilinguals extends previous findings of interference resolution to real time spoken sentence processing suggesting that bilinguals are more efficient than monolinguals at managing interference during complex sentence processing.

## Introduction

The linguistic and cognitive consequences of bilingualism have been widely investigated. Bilingualism has been associated with both slowed lexical access (e.g., Gollan et al., 2008; Ivanova and Costa, 2008; Sandoval et al., 2010) and enhanced cognitive control abilities, the ability to switch attention during information conflict (e.g., Green, 1998; Botvinick et al., 2001; Bialystok et al., 2004; Bialystok, 2005, 2007; Bialystok et al., 2009; Costa et al., 2009; Abutalebi et al., 2011). These two influences can shape language processing patterns to differ qualitatively between bilinguals and monolinguals. Cognitive consequences of bilingualism are indeed apparent in how bilinguals resolve competition during language processing when compared to their monolingual peers. In bilinguals, domain-general inhibitory control skills have been shown to relate to linguistic processing more closely than in monolinguals. A finding observed across a number of studies on phonological competition resolution while listening to words that are apparent in correlational links between linguistic processes and domain-general inhibitory control measures (e.g., Blumenfeld and Marian, 2011; Mercier et al., 2014; Giezen et al., 2015). A potentially similar pattern has emerged when bilinguals and monolinguals read garden path sentences (Teubner-Rhodes et al., 2016). However, bilingual-monolingual differences in spoken sentence-level processing have not been investigated through the lens of interference resolution. Here, we examine how bilinguals and monolinguals resolve competition that arises during complex syntactic processing while participants listen to sentences in English.

A number of studies report that monolinguals’ and bilinguals’ sentence processing are fundamentally similar, and maintain that purported differences result from inefficient lexical access routines or from an increased burden on capacity-limited cognitive resources on the part of bilinguals (Hopp, 2006, 2010, 2014; McDonald, 2006). Cunnings (2017a, b) provides critical overviews of research investigating bilingual sentence processing and argues that working memory operations that underlie sentence processing can account for similarities and differences between monolinguals and bilinguals. Rather than implicating capacity-based memory operations, he argues that the differences between monolinguals and bilinguals that persist at high bilingual proficiency levels can be characterized in terms of an increased susceptibility to interference during memory retrieval operations in sentence processing.

The role that individual differences in working memory capacity play in explaining individual differences in bilingual processing has been widely debated (Harrington and Sawyer, 1992; Juffs, 2004; Juffs and Harrington, 2011; Linck et al., 2014; Wen et al., 2015). However, recent models of sentence processing account for interference resolution during memory retrieval operations, rather than only processing capacity (Van Dyke, 2007; Van Dyke and Johns, 2012; Van Dyke et al., 2014). Specifically, mechanisms have been posited for how previously presented sentence constituents are retrieved and may trigger interference during syntactic integration in online sentence processing. Within this working memory framework, interference resolution processes have been explained (see below). How this framework relates to bilingual sentence processing remains an open question. Therefore, the motivation of this research is to compare monolinguals’ and bilinguals’ online spoken sentence processing with respect to their similarities and differences in memory operations, specifically working memory and susceptibility to interference that underlies language comprehension.

### Memory Operations Underlying Sentence Processing

There are memory-based accounts of language processing that have described sentence comprehension as involving skilled memory retrieval (e.g., Lewis and Vasishth, 2005; Lewis et al., 2006). Consider, for example, the resolution of linguistic dependencies in [1].

[1]⁢The⁢man⁢that⁢the⁢boy⁢pushes⁢has⁢a⁢red⁢shirt.

[1] is an object relative construction that involves a filler-gap dependency, in which successful interpretation requires the displaced ‘filler’ (‘the man’) to be interpreted as the direct object of the verb ‘push.’ In memory-based comprehension models, a representation of the ‘filler’ (syntactic or semantic features) will be encoded when the ‘filler’ is first encountered and then stored in memory while other words in the sentence and their representational features are processed (and themselves encoded as memory chunks). Upon reaching the verb, a syntactic operation triggers retrieval of the ‘filler’ as the direct object of the verb (during thematic role assignment). In doing so, cue-based models (e.g., McElree, 2000; McElree et al., 2003; Martin and McElree, 2008, 2009, 2011) suggest that the retrieval operation involves comparing a set of retrieval cues (lexical features) against all items in memory in parallel. The item that provides the best match to the set of retrieval cues becomes highly activated and thus retrieved. However, as the retrieval cues are matched against all items in memory in parallel, an item that partially matches the retrieval cues may sometimes be erroneously retrieved. Similarity-based interference (Gordon et al., 2001, 2004) is thus a consequence of cue-based retrieval and predicts that successful memory retrieval depends on the type of items in memory that match retrieval cues.

Cue-based models approach parsing by emphasizing the importance of the quality of representations in memory. These models predict that successful parsing depends on regulating and distinguishing these representational cues by minimizing any potential interference among them at the retrieval site during processing. Thus, the ability to detect and minimize interference among representational cues is posited to be a central aspect of complex syntactic processing. The ability to regulate lexical representations can be explained with measures that tap into susceptibility to interference in other domains that are not necessarily verbal or sentential (Van Dyke and Johns, 2012; Van Dyke et al., 2014).

The focus in cue-based models of sentence processing has thus shifted from a general-capacity model to a system that operates by detecting and managing interference. However, the mapping between parsing and the specific memory operations involved in retrieval is not well known for different syntactic structures. In the monolingual processing literature, successful garden-path reanalysis has been argued to rely on cognitive control, the ability to monitor and resolve interference, and correlates with performance on tasks that tap into conflict monitoring (Kan et al., 2013; Novick et al., 2014; Vuong and Martin, 2014). However, the memory access and revision processes required for garden-path recovery may be dissociable from the memory retrieval operations involved in the processing of linguistic dependencies in sentences such as object relatives (e.g., sentence [1] above). Thus, further research is required to systematically explain the correspondence of different memory operations to sentence parsing. The study of bilinguals relative to monolinguals provides a unique lens in this regard because of expected differences in similarity-based interference resolution between the two groups. An individual differences approach across the two groups can elucidate how working memory capacity and interference resolution skills operate during online sentence processing in each group.

### Differences in Susceptibility to an Interference Effect

In agreement with Cunnings (2017a, b), a precise characterization of how performance on memory and cognitive control tasks relates to specific aspects of sentence processing is crucial to our understanding of monolingual and bilingual sentence processing. In an extensive review, Cunnings (2017b), discusses the results of studies investigating anaphora resolution and concludes that bilingual sentence processing is more susceptible to the effects of similarity-based retrieval interference as bilinguals may differently weigh syntactic and discourse-level cues to memory retrieval. Despite these findings, there is extensive research and debate as to the positive effects bilingualism has on cognitive control. Word level and non-linguistic behavioral studies have shown that when bilinguals are matched on language skills in the shared language with their monolingual counterparts, they outperform monolinguals on tasks involving interference resolution (e.g., Bialystok, 2005, 2007; Blumenfeld and Marian, 2011). The proposed explanation for these findings is that bilinguals’ language processing requires continuous monitoring and regulation of two active mental lexicons to control for any language-related interference (Abutalebi et al., 2011). The cognitive control mechanisms that mediate cross-language conflict are known to be domain-general and are reinforced regularly due to the everyday experiences of bilinguals. Neuroimaging data have also revealed that once input has been evaluated for the presence of conflict, bilinguals outperform monolinguals in resolving the conflict and show a more efficient pattern of neural activation (namely anterior cingulate cortex) for recruiting cognitive control mechanisms to manage the conflicting information (Botvinick et al., 2001; Costa et al., 2009; Abutalebi et al., 2011). Thus, an adaptive response to the linguistic processing demands facing bilinguals shapes the way in which they leverage cognitive skills during language processing (Abutalebi and Green, 2007). Looking back to sentence level processing, in a visual sentence parsing study, Teubner-Rhodes et al. (2016) confirmed these predictions that bilinguals would resolve linguistic conflict more effectively. In this study, monolinguals’ and bilinguals’ sentence processing were compared before and after practicing two versions of a non-linguistic cognitive task, a working memory task and a cognitive control task (similar to the tasks we use in this study, see the “Materials and Methods” section). The sentence processing task was self-paced reading of garden path sentences, and bilinguals’ and monolinguals’ reaction time and comprehension accuracy during sentence processing were compared before and after the cognitive tasks had been completed. Results revealed that bilinguals and monolinguals had similar reading times and accuracy scores at the pretest (i.e., before the cognitive exercise session). However, bilinguals seemed to benefit more compared to monolinguals from the cognitive control exercise as their sentence comprehension accuracy increased post-exercise. These results suggest that the way in which bilinguals manage conflict in the non-linguistic domain may be more closely aligned with how they manage linguistic conflict, perhaps because non-linguistic skills are more routinely leveraged for linguistic processing. This pattern may have emerged because of the additional practice that bilinguals have with inhibiting irrelevant information compared to their monolingual peers (e.g., Green, 1998; Bialystok et al., 2004; Thothathiri et al., 2018; Navarro-Torres et al., 2019).

### The Current Study

The main purpose of the current study is to investigate whether bilinguals’ online processing of spoken sentences differs from monolinguals’ and, if this is the case, what the nature of that processing difference is. We predict that a key determinant of bilingual and monolingual sentence processing differences relates to both groups’ susceptibility to the effects of similarity-based interference. Here we measure monolinguals’ and bilinguals’ susceptibility to interference in their incremental processing of sentences in real time that includes syntactic dependency (similar to what is described above, [1]) using an online eye-tracking method. In processing the sentences examined here, interference arises from competition between the displaced noun phrase (‘the man’) and the intervening noun phrase (‘the boy’) during thematic licensing of the verb with its direct object (‘the man’). By utilizing an eye tracking while listening, visual world paradigm (ETL-vw), we can track the interference effect in real time, as it allows us to examine engagement and disengagement from an activated representation during processing. We define interference as an extended engagement (i.e., activation) of both NP1 and NP2 at the post-verb window, which is manifested by the overlap of gaze patterns on these NPs. In addition, interference resolution is defined as disengagement from the intervening noun phrase (NP2) and settling on the syntactically licensed direct object (NP1). Moreover, via ETL-vw, we can capture the interference effect during parsing at the individual level and then correlate it with individual differences in working memory and cognitive control processes.

In order to use demonstrated methods to measure complex working memory and cognitive control (Teubner-Rhodes et al., 2016), we test both groups on a no-conflict N-back task (a measure of working memory capacity alone) and a high-conflict N-back task (a measure of cognitive control with a working memory load similar to the no-conflict task). The high-conflict N-back task captures individuals’ moment-to-moment adjustments in control and monitoring while selecting one representation over a competing representation (i.e., lures, see details in the “Materials and Methods” section). This task activates similar neural regions as other prototypical conflict-control measures such as Stroop and flanker tasks (e.g., January et al., 2009; Ye and Zhou, 2009; Teubner-Rhodes et al., 2016), but has the additional advantage of being presented in a serial (continuous) fashion. Later, using a multilevel analysis approach, we examine how general working memory and cognitive control contribute to dependency processing across the time-course of sentence comprehension in monolinguals and bilinguals. We predict that, if bilingual-monolingual differences do exist in similarity-based interference processing, then the two groups should show differential interference effects during object relative sentence processing. Further, if bilinguals’ domain-general cognitive control skills are related to their sentence processing ability, then correlational links will be present between our linguistic and non-linguistic conflict measures.

## Materials and Methods

### Participants

Twenty-six bilingual speakers of Spanish and English (21 females, mean age = 21 years.) and 22 monolingual English speakers (17 females, mean age = 20 years.), all undergraduates from San Diego State University, participated in the study in exchange for course credit. Of these participants, 21 bilinguals (19 females, mean age = 21.6) and 19 monolinguals (16 females, mean age = 21.5) were included in the final analyses as five bilinguals and three monolinguals were excluded (see the *Analysis and screening approach* section below).

All participants completed the *Language Experience and Proficiency Questionnaire* (LEAP-Q; Marian et al., 2007) to assess their language background and current language exposure. This measure reports language learning history and different aspects of language proficiency (understanding and speaking abilities) in each of participants’ self-identified languages on a scale from 0 (*“none”*) to 10 (*“perfect”*). To meet the criteria as proficient bilingual speakers, participants had to report a score of ≥7 (*“good”*) out of 10 in each of the 3 areas for each of their languages. All bilingual participants were early learners of English and Spanish who were exposed to both languages by age 6. The bilingual group consisted of 10 simultaneous learners of English and Spanish, four native Spanish speakers, and two native English speakers. Across the bilingual group, there was no statistical difference in age of first exposure to English and Spanish [*t*(15) = 0.5, *p* > 0.05] and age of reported proficiency in English and Spanish [*t*(15) = 0.6, *p* > 0.05]. Of the 19 monolingual participants, 12 reported having learned some American Sign Language, Hebrew, French or Spanish. These ‘monolingual’ participants reported low levels of proficiency and exposure in these languages, indicating that they were not able to actively communicate in these additional languages. Thus, in this study we make the contrast between participants with self-reported extensive bilingual experience (henceforth bilinguals) and those with self-reported minimal experience with additional languages (henceforth monolinguals). In addition, basic vocabulary knowledge, attention, and general short-term memory span were assessed via the *Peabody Picture Vocabulary Test* (PPVT version 3, Dunn and Dunn, 1997), *Semantic verbal fluency* (Lezak et al., 2012), *Trail Making A/B* (Tombaugh, 2004) and *digit span* (Wechsler, 1997) tasks, respectively, to ensure participants included in analyses performed within normal limits and were matched across groups. The results of these linguistic and cognitive tests did not reveal any reliable differences between the two groups of monolinguals and bilinguals (see Table 1 for statistical results).

**TABLE 1:** Participant characteristics: Language self-reports, linguistic, and cognitive performance (19 Monolinguals and 21 Bilinguals).

Assessments	Monolingual (*n* = 19)	Bilingual (*n* = 21)		
	Mean (*SD*)	Mean (*SD*)		

Language experience and proficiency	English	English	Spanish	
Age of acquisition	0.1 (0.2)	2.1 (1.5)	← n.s. → 1.9 (1.4)	
Age of proficiency	1.1 (1.0)	3.4 (2.2)	← n.s. → 2.8 (2.7)	
Self-reported proficiency *Comprehension and speaking abilities*	9.9 (0.5)	9.9 (0.4)	← * → 8.8 (1.1)	
Percentage of exposure	100 (0)	73.8 (13.1)	26.2 (13.1)	

**Language**				***t*-test (*p*-val)**

Verbal fluency *A measure of expressive vocabulary*	54.7 (11.0)	52.7 (9.6)	35.0 (8.4)	0.6 (0.6)
Peabody picture vocabulary test (PPVT-III – Standardized score). TVIP (The Spanish form – raw score in percentages) *A measure of receptive vocabulary*	104.2 (9.2)	102.8 (10.2)	81 (8.2)	0.5 (0.6)

**Cognitive**				

Digit span: Forward *A measure of short-term memory capacity*	6.2 (0.8)	5.7 (1.1)		1.4 (0.2)
Digit span: Backward *A measure of working memory*	4.4 (1)	4.3 (0.9)		0.5 (0.6)
Trail Making Task A (seconds) *A measure of visual attention*	27.1s (7.6)	27.6 (6.9)		−0.2 (0.8)
Trail Making Task B (seconds) *A measure of visual attention and switching*	57.9s (22.2)	65.8 (16.5)		−1.3 (0.2)

The values for the PPVT-III are the standard scores where WNL is 85 and above. The values for the TVIP are percentage of correct responses computed from the raw score. Verbal fluency score was measured as the average number of exemplars produced across three semantic categories (Note that the Spanish Verbal fluency of 7 bilingual participants was missing). All other measures are within normal limits. *T*-test results are reported comparing monolinguals’ and bilinguals’ English experience and language scores, as well as cognitive scores. n.s., non-significant; *p < 0.05.

### Experimental Materials

We first describe the online eye-tracking measures followed by the memory and cognitive tasks.

#### Real-Time Sentence Processing: Eye-Tracking While Listening [ETL-vw]

**FIGURE 1:**
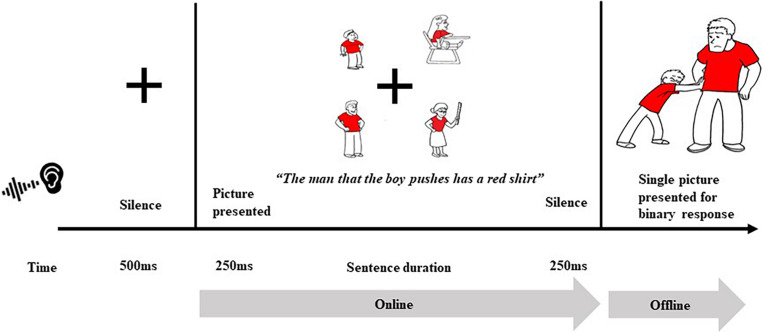
Example of an object relative condition trial in the eye-tracking while listening visual world paradigm (ETL-vw).

Participants completed an eye-tracking while listening visual world (ETL-vw) task which allowed us to capture both online processing and offline comprehension. In the online portion of the paradigm, participants were aurally presented with an uninterrupted sentence while viewing four images on the screen (see Figure 1). The offline portion of this experiment was intended to reinforce the need for the participants to attend to the sentence and measure their overall comprehension of the materials presented.

In the online portion of the ETL-vw task, a total of 80 English sentences were used, 20 experimental, 20 experimental control and 40 filler sentences. The experimental target and control items were ten-word semantically reversible object-relative (OR) and subject-relative (SR) sentences. The filler items were ten to eleven words long in the form of active (A) and passive (P) constructions:

Experimental Target (OR): The man that the boy pushes 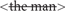 has a red shirt.
Experimental Control (SR): The man that pushes the boy has a red shirt.
Filler (A): The man is pushing the boy with a red shirt.
Filler (P): The man is pushed by the boy in the red shirt.

Half of the items were derived from the S.O.A.P. Syntactic Battery of Sentence Comprehension (Love and Oster, 2002). To increase the number of items per condition, 10 additional sentences for each of the categories were created for a total of 80 items. Sentences were recorded by a native speaker of English at a normal rate of speech (mean = 4.4 syll/sec). Appendix 1 provides the entire sentence list. As shown in Figure 1, for each sentence, a set of four images were selected that included the images of the two noun phrases (NPs) of the target sentence, NP1 (‘the man’), the displaced direct object, and NP2 (‘the boy’), the subject of the sentence, along with two distractor images of characters that had similar features (e.g., the same color of hair, shirt, pants, etc.) to the target images (‘the teacher’ and ‘the girl’). In this switched target design, these distractor images served as the target images for a different experimental sentence. This design ensures that gazes to NPs were indicative of lexical activation, and not due to a preference for a particular image. The images were sized to match one another at 450 × 450 pixels and were placed in the corner quadrants of the screen in a counterbalanced order across trials, creating four areas of interest (AOIs). Each trial began with a 500 ms fixation cross, followed by a 250 ms blank screen. The four pictures were presented 100 ms before the auditory sentence began and remained on the screen for 250 ms after the experimental sentence ended. Each trial was followed by an offline comprehension task.

In the offline portion of the ETL-vw task, each sentence was followed by a comprehension task. Specifically, 250 ms after the online portion ended, participants were presented with a picture scene with the two referents performing an action. Participants had to make a binary (YES/NO) decision if the picture they saw matched the target sentence they had just heard. The picture remained on the screen until the participant responded, or 1750 ms- whichever came first. Across the 80 trials, half the time the picture scene matched what was heard (requiring a YES response), the other half of the time, it did not (requiring a NO response). Defining modifiers (such as the color of hair, shirt, pants, etc.) were held consistent for each character in each item to prevent participants from using non-syntactic cues to identify the mentioned NPs during sentence processing.

After data collection was completed, an item analysis revealed that, in two of the experimental OR sentences, NP1 and NP2 were too similar visually. In two of the trials, we realized that the images of the sentence’s two noun phrases (e.g., the girl and the nurse) were similar to one another in terms of visual appearance. This made it hard for participants to perceptually distinguish these characters, and we expected that eye-tracking data indexing looks to NP1 vs. NP2 would be uninterpretable as a result. Thus, we decided to exclude these two items from analyses. As a result, 18 experimental and 18 control items are included in the analyses below.

#### Experimental Measure of Working Memory and Cognitive Control: N-Back

**FIGURE 2:**
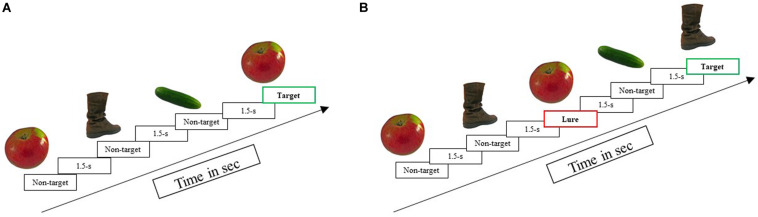
Example of design for **(A)** no-conflict N-Back: a test of working memory capacity and **(B)** high-conflict N-Back: a test of cognitive control. The green box identifies the “target” item in both **(A,B)**, while the red box identifies the “lure” item.

Participants were tested on two versions of the N-Back task: a no-conflict condition (3-back; indexing working memory capacity, Figure 2A) and a high-conflict condition [3-back; indexing cognitive control and ability to inhibit interference from lures that appeared 2, 4, or 5 (but not 3) items before the target, Figure 2B]. During the N-Back tasks, picture stimuli appeared one-by-one for 1.5-s each, with a 1.5-s inter-stimulus interval. Participants judged whether the current item presented on the screen matched the item presented three trials previously by pressing the response key for match-trials only. In each of the versions, targets comprised 50% of the trials. In the no-conflict version (Figure 2A, above), all non-match trials were non-target pictures that had not appeared before, whereas, in the high-conflict version (Figure 2B, above), 36 out of 48 non-match trials were lure items that had appeared two, four, or five trials previously (adapted from Teubner-Rhodes et al., 2016). For both, participants were instructed to respond only when an item matched a stimulus presented 3-back, which is noted by blue boxes in Figures 2A,B. The orange box in Figure 2B shows a lure condition, where participants need to inhibit a response because the item is the same as a stimulus 2 back, not 3 back. While both versions of this task involved maintaining objects in working memory, the high-conflict version additionally required participants to override their familiarity for lure items; that is, as a measure of cognitive-control skills, participants had to correctly ignore lures as non-match for the 3-back task.

### Procedure

The study protocol was approved by and the study was carried out in accordance with the recommendations of San Diego State University’s IRB committee. All participants gave written informed consent in accordance with the Declaration of Helsinki. The following assessments and experimental tasks were completed across two sessions, the order of experimental tasks was counterbalanced across participants.

Cognitive and language assessments were administered in a quiet testing room at San Diego State University. As described above, all participants completed the LEAP-Q, Peabody Picture Vocabulary Test (receptive vocabulary), Semantic verbal fluency (expressive vocabulary), Trail Making A/B (attention) and forward and reverse digit span tasks (short term memory and working memory).

For the ETL-vw task, participants were seated in front of a computer screen and a Tobii X-120 eye-tracker with their eyes at a distance of 60 cm from the eye-tracker. For all trials, gaze location was sampled at a rate of 60 Hz resulting in gaze location being recorded every 17 ms across each trial. Stimuli and button press/reaction time collection were controlled by E-prime 2.0 software (Psychology Software Tools, Pittsburgh). At the beginning of each experiment, participants completed three practice trials which allowed for questions and assurance that the participants understood the task at hand. Upon completion of the practice items, the 80 trials (40 experimental, 40 fillers intermixed) were presented. Each on-line trial was followed by an offline task in which participants responded to the comprehension questions via a binary response via a button box using their right hand.

The N-back task (no-conflict and high-conflict versions) was presented and accuracy responses were registered by E-prime 2.0 software. Responses were made via a single key press on a keyboard. Participants completed two blocks of 96 trials (1 of no-conflict and 1 of high-conflict, each starting with four practice trials).

### Analysis and Screening Approach

#### Processing ETL-vw Data

When gaze location was not available from either eye or was not within any of the areas of interest (AOIs), that gaze sample was excluded from further analysis (track loss had to be below 25% for participant inclusion in the analyses below). Next, we calculated fixations - defined as a cluster of six consecutive gazes- to denote a period where the eyes are locked on a specific AOI. Fixation proportions were computed (in 100 ms time bins) as the number of trials on which the participant was fixating each object divided by the number of trials in that condition. These proportions do not add to 1.0 because, at any given time, participants need not be fixating one of the objects – they may be looking elsewhere (e.g., screen center or off-screen), or moving their eyes (e.g., a saccade), or there may be track loss (e.g., a blink). This also means that target object fixations can increase without an arithmetically equivalent decrease in distractor object fixation, particularly early in the time course when participants are likely to be looking at the screen center until they have information that drives target fixation.

#### Individual Performance

Once fixation proportions were calculated for each participant, all data were inspected for participant’s overt use of strategy (e.g., only looking at one of the NPs throughout the sentence) and equipment (calibration) errors. Data from one monolingual participant were discarded due to fixating on the first NP throughout each of the test sentences. This strategy deviated from natural looking patterns evinced by others in that group. Data from two bilingual participants were also discarded due to equipment calibration errors making the looking patterns uninterpretable.

In addition, we reviewed individuals’ comprehension scores for each sentence type (object relatives, subject relatives, actives, and passives) and eliminated data from participants who performed below chance across all four sentence types, indicating difficulty with the task (i.e., not using both button responses, or no responses). Two monolinguals and one bilingual were excluded based on this criterion.

#### Windows of Time-Series Analyses

To account for timing differences across the sentences in the study, we coded the timing onsets for each of the syntactic constituents (i.e., onset of each noun phrase, verb, and post-verb region) across all experimental items. This allowed us to filter across sentences and examine the processing patterns at the exact windows of interest.

Recall that the goal of this study is to explore monolinguals’ and bilinguals’ differences in the way in which their language experiences shape their reaction to interference and how that is called upon during real time sentence processing. As such, we focus here on the OR constructions since only they present an environment that contains an opportunity for similarity-based interference to occur. To support this approach, below we present evidence confirming that ORs, and not SRs, produce a similarity-based interference effect (see the “Results” section: Interference resolution window). Thus, what follows is an exploration of both lexical activation and syntactically driven interference resolution during the sentence processing of OR constructions.

We have a unique opportunity to investigate lexical activation patterns for the two NPs prior to the syntactic cue for re-activation and interference resolution in the OR sentences. Here we created two windows of analyses that allowed for gaze pattern analysis within specific regions based on our hypotheses (see below).

⁢Lexical⁢access  Interference⁢resolution

[The⁢man⁢that⁢the⁢boy]⁢[pushes⁢has⁢a⁢red⁢shirt]

The timing of these windows was derived by averaging the starting and ending points for each predefined window across experimental target sentences, which resulted in a lexical access window ranging between 100 and 1200 ms (NP1: 100–600 ms and NP2: 600–1200 ms) and an interference window ranging between 1200 and 3000 ms.

The first window (hereafter, “lexical-access”) captures the presentation of the two noun phrases in the sentence (e.g., ‘the man’/‘the boy’). Looks to each picture representation of each NP upon hearing it during online comprehension are taken as measures of lexical activation. The second window (hereafter, “interference resolution”) comprises the onset of the verb until the end of the sentence. It is in this window where incoming auditory information may cause interference during processing, as both of the noun phrases are semantically allowed to serve as the theme (direct object) of the verb. Here, we argue that the lingering activation of NP2 ‘collides’ with the syntactically driven re-activation of NP1, potentially causing interference. Toward the end of this window, gaze patterns toward NP1 (the syntactically licensed direct object) should show growth over time, indicating that listeners are correctly assigning the thematic role of the theme to the fronted direct object. Real-time dependency linking is therefore gauged by either a shift in gazes to NP1 (signaling reactivation of the direct object) or an increase in gazes to NP1 if there was a gaze preference for NP1.

#### Growth Curve Analysis

The fixation time course data were analyzed using Growth Curve Analyses (GCAs) with second-order orthogonal polynomials (the intercept is included by default), which is a multi-level modeling technique specifically designed to capture change over time (Mirman et al., 2008; Dink and Ferguson, 2015). We tested the reliability of the difference between NP1 and NP2 at each of the windows of analysis using this approach (GCA; Mirman, 2016). Effects of the variables of interest (group, cognitive skills) on the polynomial terms provide a way to quantify and evaluate those effects on statistically independent (i.e., orthogonal) aspects of the fixation proportions trajectory. In the GCA approach, the Level 1 model captures the overall fixation time course with the intercept term reflecting average overall fixation proportions, and the linear term reflecting a monotonic change in fixation proportion or slope (Mirman et al., 2008). All analyses were conducted in the statistical software R-3.2.1, using the package LmerTest.

#### N-Back Tasks

Performance accuracy for both versions of the N-back tasks was scored using *d*’ prime to account for errors of omission (missed targets) and commission (false hits, Gaetano et al., 2015). A higher *d*’ indicates that participants were better able to perform the task (perfect performance in this study would have yielded a *d*’ of ∼4). As detailed below, we compared participants’ *d*’ scores across condition (high-conflict vs. no-conflict) and group (bilingual vs. monolingual) using a *t*-test to examine if we could replicate previous findings of bilinguals outperforming monolinguals in complex tasks that demand cognitive control (e.g., Teubner-Rhodes et al., 2016). As mentioned above with the ETL-vw data, data from participants were excluded from further analysis if they demonstrated a lack of understanding of the task/materials or for equipment error. For the N-back task, data from two participants in the bilingual group were discarded due to equipment error, leaving a total number of 21 bilinguals and 19 monolinguals in the final analyses.

## Results and Discussion

### Offline Comprehension

We examined offline sentence comprehension accuracy to ensure that participants were able to understand the task and sentences. Our groups did not differ in their rate of comprehension success. Recall that after each on-line sentence processing trial, participants were shown a scene and were asked if the picture matched what they just heard (via a binary button press response, see Figure 1 above). Across all sentence types, monolinguals (*M* = 70%, *SD* = 10, *n* = 19) and bilinguals (*M* = 70%, *SD* = 10, *n* = 21) showed similar performance [*t*(38) = 0.04, *p* = 0.9]. A similar pattern held for the OR sentences (which are the focus of subsequent analyses), with monolinguals (*M* = 65%, *SD* = 10) and bilinguals (*M* = 60%, *SD* = 20)^1^ performing similarly [*t*(38) = 0.7, *p* > 0.5]. In terms of their reaction times, monolinguals (*M* = 926.7.4 ms, *SD* = 100.4) were slightly slower than bilinguals (*M* = 884.1 ms, *SD* = 116.7) across all sentence types; however, this was not statistically significant [*t*(38) = 1.2, *p* > 0.5]. A similar pattern held for the OR sentences, with monolinguals (*M* = 887.4 ms, *SD* = 131.3) and bilinguals (*M* = 840.6 ms, *SD* = 186.9) performing similarly [*t*(38) = 0.8, *p* = 0.4]. This suggests that bilinguals and monolinguals performed on par with one another in their offline comprehension of sentences.

### Time-Course of Online Sentence Processing

We present the gaze pattern data for the critical experimental sentence condition (OR), which allows for an investigation of both lexical access and syntactic dependency linking, with a focus on interference.

**FIGURE 3:**
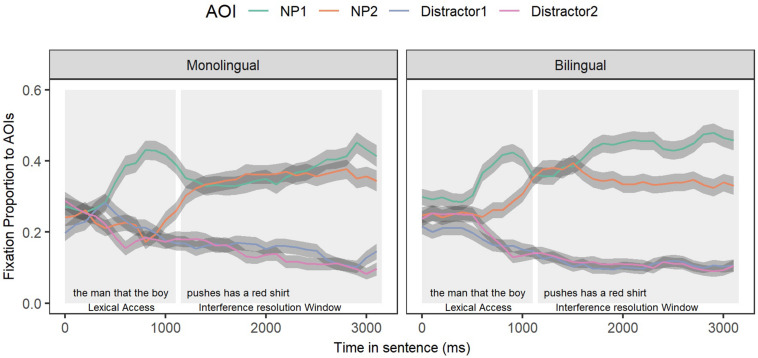
Proportion of looks to each area of interest (AOI) in OR sentences for bilinguals **(right panel)** and monolinguals **(left panel)**. The first window represents lexical access; the second window represents interference resolution. Error bars (shaded areas) are within-subject 95% confidence intervals.

In the ETL-vw task, upon initially hearing each noun phrase (NP), listeners looked at the correct picture representation of that NP (i.e., upon hearing ‘the man’, looks increased toward the picture of ‘the man’). Figure 3 depicts the time course of fixations to NP1 (green line) and NP2 (orange line) and the two other unrelated noun phrases (Distractor 1- purple line and Distractor 2- pink line) across groups for OR sentences across all trials. First, we observed a clear divergence of looks to the relevant picture representations of the mentioned NPs (NP1 and NP2) from those pictures that were not mentioned (looks to the unrelated items were below chance, 25%). This divergence is demonstrated early in the sentence and remains as the sentence unfolds. Since participants did not look at the irrelevant picture options, we focus the rest of our analyses on the two critical NPs of interest (NP1 and NP2). To understand differences across monolingual and bilingual participants, as mentioned above, we identified windows of analysis to capture different levels of online sentence processing; lexical access (100–1200 ms), where listeners were expected to gaze to the correct picture representation of the NPs they heard and the interference window (1200–3000 ms), which captures dependency processing, where listeners were expected to re-activate the displaced NP (‘the man’) after processing the verb (‘pushes’). Below, we show analyses for both windows and the differences that are depicted based on the groups.

#### Lexical Access Window

As previous studies have reported differences in lexical access patterns across bilinguals and monolinguals, we started by comparing groups’ performance in lexical access (again, between 100 and 1200 ms) for NP1 (100–600 ms) and NP2 (600–1200 ms) during sentence processing for OR sentences.

##### Group differences in lexical access

To compare the rate and extent of NP1 and NP2 activation across groups, we conducted GCAs (see Table 2 for statistical results). For this analysis, the Level 2 model contained the group (monolingual versus bilingual). The results of these analyses were not significant, meaning that the level and rate of NP1 activation over time were similar across groups (NP1: Intercept *Estimates* = 0.01, *SE* = 0.03, *p* = 0.7; Linear *Estimates* = −0.05, *SE* = 0.03, *p* = 0.1). Similarly, when hearing the second NP, the difference between the groups was not significant on the linear term (*Estimates* = 0.06, *SE* = 0.04, *p* = 0.13), suggesting equivalent rates of lexical activation. However, bilinguals had an overall higher proportion of gazes toward NP2 than monolinguals, which appears to reflect greater NP2 activation (Intercept *Estimates* = 0.07, *SE* = 0.02, *p* < 0.05). While the latter is an intriguing result, it does not change the core finding that the bilinguals tested here are *not* at a disadvantage for rate of lexical activation during sentence processing. We can thus confirm that while processing OR sentences there was no difference between monolinguals and bilinguals for the rate of lexical activation over time, a measure of speed of lexical access.

**TABLE 2:** Results of the GCA analysis examining group differences in lexical access for NP1 and NP2.

	NP1				NP2			
*Fixed effects*	Estimates	SE	*t*	*p*-value	Estimates	SE	*t*	*p*-value
Intercept	0.27	0.05	5.12	<0.001	0.15	0.04	4.03	<0.001
Linear term	0.11	0.06	2.02	0.05	–0.03	0.06	–0.52	0.06
Group	0.01	0.03	0.33	0.74	0.07	0.02	2.96	0.01
Group*Linear term	–0.05	0.03	–1.60	0.11	0.06	0.04	1.54	0.13

***Random effects***								
**Subject**	**Variance**				**Variance**			

Intercept	0.008				0.004			
Linear term	0.001				0.004			

#### Interference Resolution Window

**FIGURE 4:**
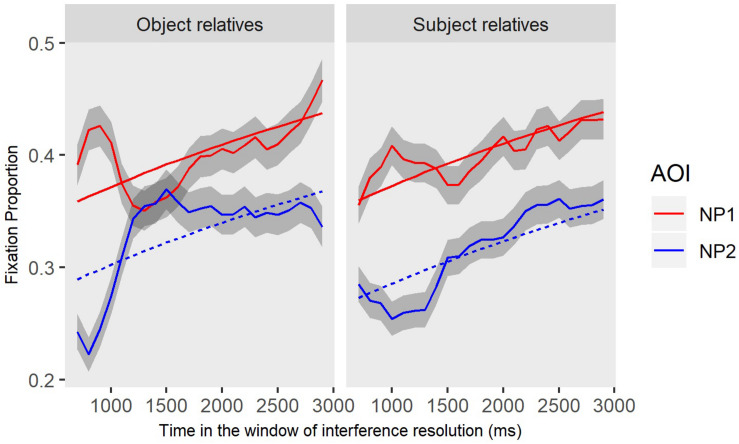
Gaze patterns (observed and GCA model fit) toward NP1 and NP2 in interaction with sentence type (object relative [OR] vs. subject relative [SR]). At the region that the thematic role assignment should happen for both OR (…the boy pushes has a red shirt) and SR (…pushes the boy has a red shirt) sentences, we only observed an interference effect for OR sentences, as depicted by overlap between NP1 and NP2. Shaded areas represent 95% confidence intervals and lines of best fit for the statistical model are included.

We first established the predicted interference effect within object relative sentences. Looking at the OR sentences (Figure 3), during the interference resolution window, it is expected that the direct object NP1 (‘the man’) will be reactivated after processing the verb (‘push’). If there is no interference (between NP1 and NP2) in assigning a thematic role of theme to NP1 during the dependency linking process, then we expect re-activation to be indicated only by increases in looks to NP1 as compared to NP2. However, if there is interference in the linking of the direct object to the verb, then we expect to see overlapping (or equal) looks to NP1 and NP2. We first validated that interference between NP1 and NP2 is specific to OR processing demands. To do so, we compared OR gaze patterns to those for SR (experimental control) sentences. As shown in Figure 4, the mutual period at which the thematic role assignment is expected to happen in both sentence constructions is a time window between 700 and 3000 ms, which is the post copula region in both sentence types (e.g., ‘… pushes has a red shirt’). This time window reflects the occurrence of thematic role assignment in both of the sentence types.

To capture the differences in processing demands for SR and OR sentences, we conducted GCAs (see Table 3 for statistical results) examining the sentence types. For this analysis, the Level 2 model contained the interactions between AOIs (NP1: red line versus NP2: blue line) and sentence types (OR: left panel versus SR: right panel) across all participants. The interaction effect was significant, revealing significantly more overlap (i.e., interference) between NP2 and NP1 in OR sentences as compared to SR sentences at the intercept level (*Estimates* = −0.02, *SE* = 0.007, *p* = 0.01). Note that the parameter estimates were for NP2 (intervener) relative to NP1 (displaced object), so a negative estimate for the intercept term corresponds to a smaller interference effect for SR sentences. In other words, the proportion of gazes to the intervening NP2 after the verb is heard was higher in OR compared to SR sentences. Remember that in both of these sentence types the activation of NP2 is expected given that participants had just heard and processed it in the ongoing sentence. Of interest here in the OR sentences is the fact that continued NP2 activation might interfere with the syntactically driven reactivation of NP1 when the verb is processed. A greater and extended overlap between NP1 and NP2 for the OR sentences represents our definition of interference, which is not observed in the SR sentences. Since we are interested in exploring interference and its resolution between monolinguals and bilinguals, the rest of the analyses focus on the OR data.

**TABLE 3:** Results of the GCA analysis examining evidence of interference in OR sentences compared to SR sentences.

*Fixed effects*	Estimates	SE	*T*	*p*-value
Intercept	0.5	0.008	59.38	<0.001
AOI	–0.07	0.005	–13.22	<0.001
Sentence type	0.002	0.005	0.31	0.80
AOI*Sentence type	–0.02	0.007	–2.43	0.01

***Random effects***				
**Group**	**Variance**			

Intercept	0.003			
Linear term	0.013			

##### Group differences in interference resolution

**FIGURE 5:**
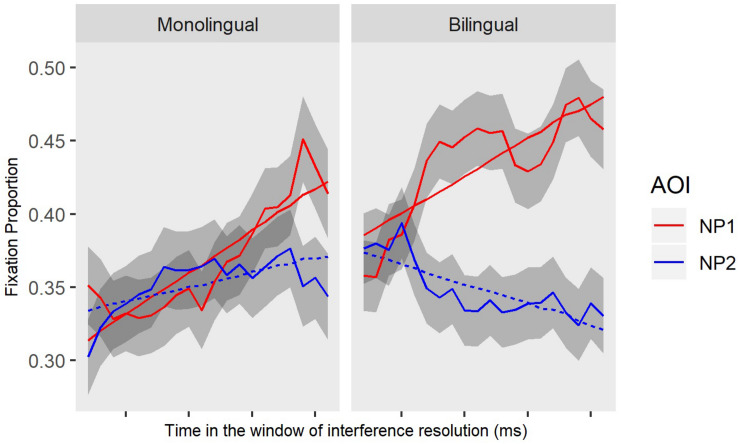
Gaze patterns (observed and GCA model fit) toward the displaced object (NP1) and the intervener (NP2) in interaction with group (monolingual vs. bilingual) in OR sentences. The interference window starts at verb onset through the copula “has.”

We next examined whether monolinguals and bilinguals differ in real time dependency linking after the initial lexical access stage. For this analysis, we built a baseline model with the fixed effect of AOI (NP2 versus NP1) on the linear time term, and also a full model by adding the group variable (monolingual versus bilingual) into the interaction. Improvements in model fit were evaluated using 2 times the change in log-likelihood, which is distributed as *x*^2^ with degrees of freedom equal to the number of parameters added. The data and model fits are shown in Figure 5, with the monolingual group in the left panel and the bilingual group in the right panel. Note that the parameter estimates were for NP2 (intervener) relative to NP1 (displaced object), so a negative estimate for the intercept term corresponds to a smaller interference effect or better competition resolution. The results of the full model (see Table 4 for statistical results) revealed that group is predictive of the NP2 versus NP1 pattern at the intercept level (*Estimates* = −0.05, *SE* = 0.01, *p* < 0.05), meaning that on average bilinguals had a significantly larger gap between NP2 and NP1 as compared to monolinguals (i.e., less overlapping gaze patterns, meaning less interference). Importantly, this interaction effect was observed at the linear term (*Estimates* = −0.07, *SE* = 0.04, *p* < 0.05), meaning that as a function of time the gap between NP2 and NP1 became larger in the bilingual group. This indicates faster resolution of interference in the bilinguals compared to the monolinguals. Recall that both of these processing patterns across groups yielded similar comprehension accuracy and offline comprehension latencies; however, there are significant differences in the two groups’ susceptibility to interference caused by the intervening NP2 during real time processing.

**TABLE 4:** Results of the GCA analysis examining differences in interference effects between the groups in OR sentences.

*Fixed effects*	Estimates	*SE*	*t*	*p*-value
Intercept	0.40	0.01	43.47	<0.001
AOI	–0.05	0.01	–8.77	<0.001
Linear term	0.13	0.02	6.62	<0.001
Group	0.04	0.01	3.44	<0.05
AOI*Linear term	–0.01	0.03	–5.72	<0.001
AOI*Group	–0.05	0.01	–6.07	<0.001
Group*Linear term	–0.03	0.03	–0.44	0.66
AOI*Group*Linear term	–0.07	0.04	–1.99	0.04

***Random effects***				
**Subject**	**Variance**			

Intercept	0.003			
Linear term	0.004			

Overall, we found that there is a difference in the dependency processing patterns of monolinguals and bilinguals. The question that remains is what the underlying source of this difference is. As Table 1 suggests, individuals across each group are matched in their language competency, short term memory skills, English vocabulary, semantic fluency, and attention. Of interest, here is whether or not other behavioral sources could account for the processing differences between monolinguals and bilinguals. Below, we compare groups on their performance on working memory and cognitive control and build models to examine whether these systems can explain the observed processing differences at the sentence level between the two groups.

### Behavioral Cognitive Differences: N-Back Performance

Using *d*’ (*d* prime), we measured individuals’ performance on the N-back task in two conditions: high-conflict and no-conflict which are indexing cognitive control and working memory, respectively. A higher *d*’ indicates that participants were better able to perform the task (perfect performance in this study would have yielded a *d*’ of ∼4). No performance differences were observed between monolinguals and bilinguals in either of the two N-back conditions. Thus, we did not observe a general benefit in performance for bilinguals across high-conflict [*t*(38) = 1.4, *p* = 0.2] or no-conflict [*t*(38) = 0.3, *p* = 0.7] trials. While there are no group differences, we asked if these aspects of cognitive processes have any predictive value for the processing differences seen between monolinguals and bilinguals at the sentence level. Of interest is whether monolinguals and bilinguals rely differently on their cognitive control skills during the processing of complex sentences.

### Memory Operations During Sentence Processing

During the processing of OR sentences, bilinguals showed less susceptibility to interference when compared to monolinguals, as indicated by faster interference resolution evident in a wider gap between NP2 relative to NP1 (Figure 5). Here we examined the extent to which the experimental measures of no-conflict (working memory) and high-conflict N-back (cognitive control) modulated the resolution of this interference effect during parsing. We also examined whether these individual differences measures played similar or distinct roles across monolinguals and bilinguals.

**FIGURE 6:**
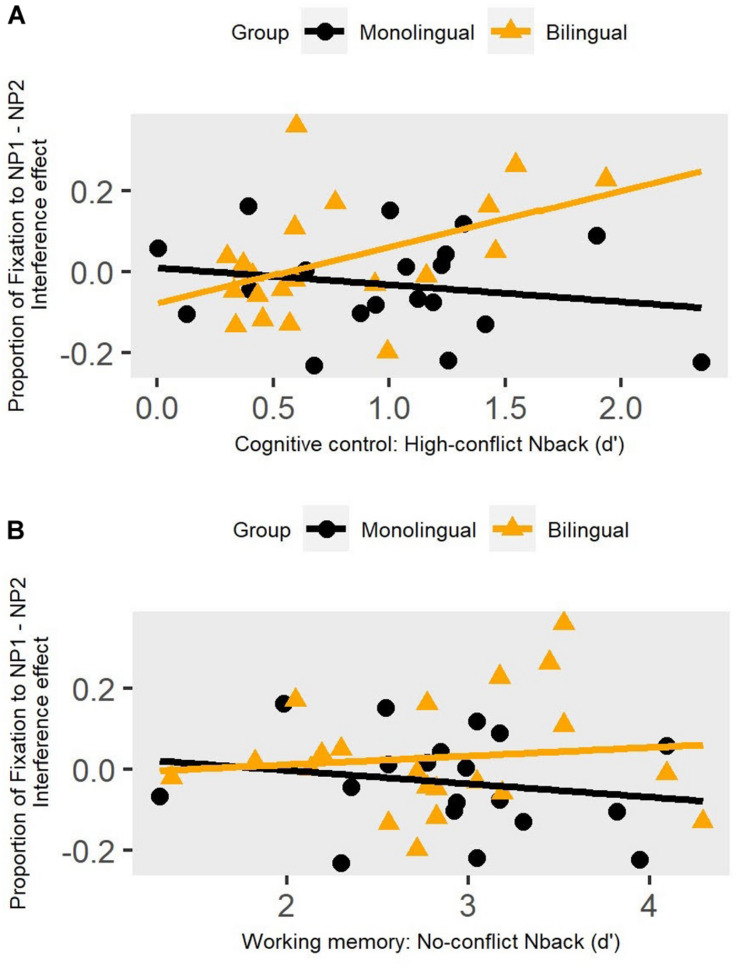
Regression fit between the size of NP1 and NP2 overlap (indicating individual differences in interference resolution) in the eye tracking task (looks to the displaced NP versus the intervening NP) and the two measures of N-back tasks. The top panel **(A)** shows the regression with the magnitude of cognitive control (high-conflict). The bottom panel **(B)** shows the regression with working memory (no-conflict).

Growth curve analysis models can account for individual participant random effects. For each individual, the magnitude of interference was calculated as the average fixation proportion on the intervening NP2 minus the displaced NP1 in the interference resolution window. Please note that, in contrast to the proportions calculated above, for this analysis we subtracted NP2 from NP1 (= NP1 – NP2) to get a positive value for ease of interpretation (i.e., larger values represent better interference resolution). The difference between intervening and displaced object (NP1-NP2) random effects for each participant can be used as a GCA measure of effect size for each participant. The extent to which each individual is different from the mean model term for each object type (intervening versus displaced) was quantified by random effects for a given model term. This was achieved based on individual-by-AOI-type random effects from a growth curve model that included no group fixed effects. Here we examined the random effects on the intercept term. The effect sizes of participants varied considerably in their fixation proportions to the intervening NP2 in this window, showing variance in ability to inhibit looks to the intervening NP2. We then asked if the effect sizes in the eye-tracking and N-back tasks were correlated across groups. Figure 6 shows the regression between cognitive control and sentence-level interference resolution (Figure 6A), as well as working memory and sentence-level interference resolution (Figure 6B) for monolinguals (black line) and bilinguals (orange line) separately. The two variables were themselves not correlated (*r* = 0.2, *p* = 0.2), so they were both entered as regressors in a GLM with the magnitude of interference as the dependent variable. Only cognitive control was reliably predictive of the sentence-level interference effect, and this was true only in the bilingual group, as indicated by significant interaction results (cognitive control: *Estimates* = 0.2, *SE* = 0.07, *p* = 0.01; working memory: *Estimates* = 0.03, *SE* = 0.05, *p* = 0.5; model’s *R*^2^ = 0.20)^2^.

## General Discussion

In an eye-tracking while listening study, we compared monolingual and bilingual individuals’ real time processing of object relative sentences. These sentences contain syntactic dependencies where two noun phrases must be assigned thematic roles once the verb is reached. We found that compared to monolinguals, bilinguals were less susceptible to the effect of similarity-based retrieval interference during syntactic integration. In particular, we found that a key determinant of bilingual processing differences related to variability in cognitive control skills, which is the ability to determine and inhibit interfering information during processing. Specifically, bilinguals who showed more efficient interference resolution on a high-conflict N-back task were also found to show smaller interference effects at the sentence level. Together, findings from the lexical activation and interference resolution stages of auditory sentence processing suggest that bilinguals and monolinguals show highly similar time-courses of activation and processing when listening to sentences in their most proficient language. In addition, group differences in sentence-level interference resolution suggest cognitive-linguistic differences across the two groups.

### Interference During Syntactic Dependency Linking: Eye-Tracking

Dependency-linking theories assume that words and phrases are encoded in some form of memory and that processing a verb triggers a search in memory or retrieval for a noun that has certain representational cues to be linked with the verb. Cues can be drawn from the properties of lexical items, including pragmatic features, morphosyntactic agreement features, or local syntactic and semantic context. These features consist of relational information between items in memory (Alcocer and Phillips, 2012; Kush, 2013). When a search is carried out in memory using a set of retrieval cues, increased processing difficulty is observed when multiple nouns have the features that match the retrieval cues to be integrated with the verb. The similarity between these featural cues has been argued to result in a similarity-based interference effect (Gordon et al., 2004, 2006). Cue-based models show that successful comprehension depends upon the efficient use of retrieval cues to distinguish target items from a field of distractors, which could be highly related to the target on various dimensions. In other words, the parser must be susceptible to and resolve interference. In a similar vein, in processing the experimental sentences that were used here [1], both of the NPs (‘the man’ and ‘the boy’) can be considered as the direct object of the verb (‘pushes’). The overlapping similarities in lexical properties result in interference between the displaced direct object (‘the man’) and the intervening noun phrase (‘the boy’). When arriving at the post verb phrase (‘has a red shirt’), correct sentence interpretation entails that listeners settle on the correct NP as the direct object during real time parsing, indicating that ‘the man was pushed.’ In this study, our goal was to examine differences in bilinguals’ versus monolinguals’ reaction to interference during the real time parsing of OR sentences. We found that bilinguals disengaged from the intervening NP earlier than monolinguals, as indexed by an earlier divergence of looks to NP1 versus NP2 following the interference effect.

Our current findings differ from the results reported by Cunnings (2017b; and references therein). Cunnings et al. (2017) suggest that L2 sentence processing is more susceptible to the effects of similarity-based retrieval interference. In this review, Cunnings discusses results from studies investigating anaphora resolution (Felser, 2016; Cunnings et al., 2017) and concludes that the increased susceptibility to interference results from L1 and L2 speakers differently weighting syntactic and discourse-level cues to memory retrieval. Specifically, as late L2 listeners may rely less on syntactic cues, they come to rely more on discourse-level and semantic cues, making them more susceptible to similarity-based interference. The findings from Cunnings (2017b) provide an important context for interpretation of the current findings: They suggest that bilingual proficiency, language learning history (L1 vs. L2) and linguistic context may modulate similarity-based interference effects.

We note that our study is different from those discussed in Cunnings (2017b) in a number of ways. First, our stimulus sentences are neutral in terms of semantic and discourse contents. As such, our stimuli do not provide a context to distinguish syntactic from discourse or semantic cues. Second, our participants are early learners of English with English being their most proficient language. Accordingly, our bilingual and monolingual groups were matched on their sentential comprehension performance, receptive, and expressive vocabulary skills in English. The bilingual group in this study would be expected to be equally sensitive to syntactic cues as their monolingual counterparts, and this was confirmed by our findings.

The current bilinguals’ status as highly proficient English listeners is also apparent in the time-course of NP1 and NP2 activation. No differences were observed between bilinguals and monolinguals in terms of slope of initial lexical activation. When bilinguals listen to single words in a less proficient language, delays in lexical activation have been identified relative to more proficient bilinguals and monolinguals (Blumenfeld et al., 2013). Such a pattern may also be expected at the sentence level when bilinguals listen in a less proficient language. Indeed, the robustness of lexical representations is a critical factor for comprehension during parsing. Decades of memory research (e.g., Dosher, 1976, 1981; McElree and Dosher, 1989) have established that the probability of retrieving particular items during the dependency-linking process relies on the strength or distinctiveness of the lexical representation itself. Here we did not find a general difference between the bilinguals and monolinguals on their level of activation of the lexical items within the sentences. Thus, our findings on the differences between the dependency processing patterns cannot relate to the extent of activation of to-be-retrieved lexical representations at the gap site.

### Nature and Engagement of Cognitive Control Skills

We then asked whether the processing difference identified in bilinguals relative to monolinguals stemmed from a difference in cognitive abilities between our groups. Bilingualism has been argued to act as a form of cognitive control training, bestowing measurable advantages in conflict monitoring – the ability to detect unpredictable conflict and flexibly adjust recruitment of cognitive control resources (Bialystok et al., 2004, 2009; Martin-Rhee and Bialystok, 2008; Costa et al., 2009; Bialystok, 2010). Such domain-general cognitive control skills could be involved in handling the similarity-based interference effect during sentence parsing. However, differences between young monolinguals and bilinguals in non-verbal cognitive control have not been found across many studies (e.g., Bialystok et al., 2005; Blumenfeld and Marian, 2011; Paap and Greenberg, 2013; Gathercole et al., 2014; Giezen et al., 2015; Freeman et al., 2017; other studies of this nature may have remained unpublished due to publication bias, De Bruin et al., 2015; also see Kroll and Bialystok, 2013 for discussion). In fact, when we examined our groups on different measures of cognitive tasks (cognitive control and working memory), monolinguals and bilinguals performed similarly as has frequently been the case for college-aged participants.

Despite similarities in bilinguals’ and monolinguals’ cognitive control and working memory skills, we found that the two groups differed in how their cognitive control skills were related to sentence processing patterns. Specifically, bilinguals who showed better cognitive control skills on the N-back task also showed overall smaller interference effects at the sentence level. However, monolinguals with higher N-back performance did not show any indication of reduced similarity-based interference effects during sentence processing. It is thus possible that bilinguals’ smaller similarity-based interference effects at the sentence level can be tied to their leveraging of cognitive control skills in this linguistic context. It has been argued that bilinguals’ use of cognitive control during sentence processing is an adaptive response to a greater level of interference during language processing, since interference can occur both within-language (as is the case here) and between-language (e.g., Abutalebi and Green, 2007). This finding is in line with research showing that bilinguals may recruit and depend on cognitive resources more than monolinguals do during interference resolution at the single word level (e.g., Blumenfeld and Marian, 2011; Mercier et al., 2014; Giezen et al., 2015) and at the sentence level (Teubner-Rhodes et al., 2016; Thothathiri et al., 2018). With the current findings, we extend this emerging pattern in the literature to a novel linguistic processing context, OR sentences. Future work is needed to further detail the link between non-linguistic cognitive control skills and sentence processing abilities in bilinguals. It is possible that bilinguals do not directly engage the non-linguistic cognitive control skills indexed here, but rather that a mediating mechanism links the two. At present, it can only be concluded that, in bilinguals, conflict resolution in sentence processing is in some manner linked with domain-general cognitive skills.

The more extended similarity-based interference that we observed during OR sentence processing for the monolingual relative to the bilingual group should not be interpreted as a noisy or inefficient parsing pattern. These participants were similarly successful in comprehending the offline question. In fact, monolingual’s extended interference effect has a non-significant yet a positively correlated pattern with the working memory measure (as indexed by the no-conflict N-back task): monolingual individuals with better working memory abilities trended toward a more extended interference effect. Thus, the differences between the groups that we have identified here suggests that language experience (bilingual or monolingual) can shape how the parser handles similarity-based interference. In this, multiple processing pathways may lead toward the same outcomes in terms of offline comprehension in proficient English speakers. Future research is needed to expand these findings to different sentence processing contexts and to further examine how extent and nature of bilingual experience may shape processing. Bilingual participants in the current study were unbalanced Spanish speakers, with stronger dominance in English than Spanish. It is possible that findings would look somewhat different when more Spanish-dominant or language-balanced bilinguals were tested in English. This is especially in light of findings that bilingual experience may shape cognitive control (Goral et al., 2015). Future work can further examine such patterns. This is an initial study to investigate monolingual-bilingual differences in the relationship between cognitive skills and processing of object relatives. Future studies should further investigate this question by taking into account additional aspects of bilingualism such as language usage and language proficiency (i.e., frequency of code switching) that can affect cognitive functioning, and by using larger sample sizes to replicate the effects observed here.

In sum, in this paper, our aim was to identify how multiple cognitive processes coordinate with one another to support bilinguals’ and monolinguals’ language processing in real time. In this regard, we found that for bilinguals, but not monolinguals, the magnitude of cognitive control (as measured by a high-conflict N-back task) was associated with the ability to resolve interference in our real time auditory sentence processing task. Specifically, bilinguals with better inhibitory control skills on the high-conflict N-back task also showed smaller interference effects at the sentence level. We did not find this association with the working memory measures (no-conflict N-back, backward digit span). Here we do not argue that the processing difference indicates an advantage for the bilingual group, instead such differences reflect adaptive changes in cognitive control as a function of learning and using language in a competitive processing domain (i.e., in a bilingual context where linguistic competition is present both within-language and between-language, Abutalebi and Green, 2007).

## Data Availability Statement

The datasets generated for this study are available on request to the corresponding author.

## Ethics Statement

The studies involving human participants were reviewed and approved by the San Diego State University. The participants provided their written informed consent to participate in this study.

## Author Contributions

All authors contributed to the conceptualization and design of the current study. NA collected and analyzed the data. All authors contributed to interpretation of findings and writing of the manuscript.

## Conflict of Interest

The authors declare that the research was conducted in the absence of any commercial or financial relationships that could be construed as a potential conflict of interest.

## Footnotes

1 We note that the comprehension accuracy for ORs was lower than in prior reports in the literature. We believe this is simply a by-product of the offline task, which was structurally different than is common in standard sentence-picture matching tasks.

2 A similar confirmatory analysis was done using backward digit span as an alternative working memory measure. This analysis was also not significant.

## Appendix

**TABLE A1:** Sentence materials used in the study as the experimental control and target. The sentences were presented in randomized order.

Experimental control – Subject relative	Experimental Target – Object relative
The cowboy that captures the Indian has blue pants.	The cowboy that the Indian captures has blue pants.
The man that grabs the boy has brown hair.	The man that the boy grabs has brown hair.
The soldier that examines the boy has black hair.	The soldier that the boy examines has black hair.
The teacher that instructs the boy has a blue shirt.	The teacher that the boy instructs has a blue shirt.
The soldier that questions the doctor has blonde hair.	The soldier that the doctor questions has blonde hair.
The man that pushes the boy has a red shirt.	The man that the boy pushes has a red shirt.
The man that records the woman has brown hair.	The man that the woman records has brown hair.
The doctor that accuses the patient has black hair.	The doctor that the patient accuses has black hair.
The boy that chases the girl has a green shirt.	The boy that the girl chases has a green shirt.
The boy that hugs the woman has a green shirt.	The boy that the woman hugs has a green shirt.
The child that kisses the woman has blonde hair.	The child that the woman kisses has blonde hair.
The boy that hits the man has a red shirt.	The boy that the man hits has a red shirt.
The boy that bites the girl has a yellow shirt.	The boy that the girl bites has a yellow shirt.
The girl that caresses the nurse has blonde hair.	The girl that the nurse caresses has blonde hair.
The woman that visits the man has blonde hair.	The woman that the man visits has blonde hair.
The girl that applauds the teacher has a red skirt.	The girl that the teacher applauds has a red skirt.
The man that punches the policeman has black hair.	The man that the policeman punches has black hair.
The girl that pinches the boy has brown hair.	The girl that the boy pinches has brown hair.
